# The Harmonic Scalpel versus Conventional Hemostasis for Neck Dissection: A Meta-Analysis of the Randomized Controlled Trials

**DOI:** 10.1371/journal.pone.0132476

**Published:** 2015-07-10

**Authors:** Zhen-Hu Ren, Jian-Lin Xu, Teng-Fei Fan, Tong Ji, Han-Jiang Wu, Chen-Ping Zhang

**Affiliations:** 1 Department of Oral and Maxillofacial & Head and Neck Oncology, Shanghai Ninth People’s Hospital, Shanghai Jiao Tong University School of Medicine, Shanghai, China; 2 Department of Respiratory Medicine, Shanghai chest hospital, Shanghai Jiao Tong University School of Medicine, Shanghai, China; 3 Department of Oral and Maxillofacial Surgery, Hospital of Stomatology, Wuhan University, Wuhan, China; 4 Department of Oral and Maxillofacial Surgery, The Second Xiangya Hospital of Central South University, Changsha, China; School of Medicine, Fu Jen Catholic University, TAIWAN

## Abstract

**Objective:**

Neck dissection is the most definitive and effective treatment for head and neck cancer. This systematic review aims to compare the efficacy and surgical outcomes of neck dissection between the harmonic scalpel and conventional surgical techniques and conduct a quantitative meta-analysis of the randomized trials.

**Methods:**

Randomized controlled trials (RCTs) were identified from the major electronic databases (MEDLINE, **EMBASE** and Cochrane Library) using the keywords ‘‘harmonic scalpel’’ and ‘‘neck dissection,’’ and a quantitative meta-analysis was conducted. The operative time and intraoperative bleeding were the primary outcome measures, and other parameters assessed included the drainage fluid volume and length of hospital stay.

**Results:**

Seven trials that met the inclusion criteria included 406 neck dissection cases (201 in the harmonic scalpel group). Compared with conventional surgical techniques, the HS group had an operative time that was significantly reduced by 29.3 minutes [mean difference: -29.29; 95% CI = (-44.26, -14.32); P=0.0001], a reduction in intraoperative bleeding by 141.1 milliliters [mean difference: -141.13; 95% CI = (-314.99, 32.73); P=0.11], and a reduction in drainage fluid volume by 64.9 milliliters [mean difference: -64.86; 95% CI = (-110.40, -19.32); P=0.005] , but it is not significant after removal of studies driving heterogeneity. There was no significant difference in the length of the hospital stay [mean difference: -0.21; 95% CI = (-0.48, 0.07); P=0.14].

**Conclusion:**

This systematic review showed that using the harmonic scalpel for neck dissection significantly reduces the operative time and drainage fluid volume and that it is not associated with an increased length of hospital stay or perioperative complications. Therefore, the harmonic scalpel method is safe and effective for neck dissection. However, the statistical heterogeneity was high. Further studies are required to substantiate our findings.

## Introduction

Head and neck cancer (HNC) accounts for approximately 6% of all human cancers. Approximately 47,560 new cases in the USA and at least 500,000 cases occur each year worldwide [1]. Neck dissection plays an important role in the treatment of HNC and is an indispensable part of many HNC treatments. There are several important anatomical structures in the neck. Injury of these vital structures could result in many postoperative complications such as hematoma, wound infection, chylous leakage, dysphagia and other issues [2, 3]. In addition, several studies have demonstrated that blood loss and the operative time are relevant for the clinical outcomes and the postoperative complications [4, 5]. The longer operative time and transfusion of erythrocytes are related to the prolonged hospital stay [6]. Therefore, many head and neck surgeons have been trying to reduce surgical complications, not only through novel surgical devices but also through new surgical approaches [7–10].

New surgical devices and technologies that focus on reducing operative time, blood loss, and other complications have been investigated and the results have been favorable [8]. The harmonic scalpel (HS) was introduced in the early 1990s and has 4 functions during the surgery: cutting of the tissues, cavitation, coaptation of the tissues and coagulation. The actions of the HS on the tissues are changed by varying the energy levels or tissue tension. Specifically, faster cutting and less hemostasis are achieved with a higher power level, more hemostasis and slower cutting are achieved with a lower power level, and with a higher tension on the tissues, the cutting is quicker. The history of the HS in head and neck surgery is brief. The most prominent advantages of the HS in head and neck surgery are that the surgical field remains bloodless, greatly reducing operative time. There have been many studies demonstrating that the HS reduces intraoperative bleeding and/or the operative time in many types of head and neck surgery such as glossectomy, submandibular gland resection, superficial parotidectomy, thyroidectomy, tonsillectomy, resection of oral cavity tumors, rhytidectomy, and the surgical treatment of rhinophyma [11–13]. However, whether the HS can reduce the operative time and intraoperative bleeding in neck dissection remains controversial [11, 14].

The purpose of this article is to review the literature (which compares the outcomes of the harmonic scalpel and traditional hemostasis in neck dissection) and conduct a meta-analysis of the randomized control trials that compare these surgical techniques (e.g., the classical technique of tying and knots and bipolardiathermy).

## Materials and Methods

### Literature search

A computerized, systematic literature search was performed using MEDLINE, EMBASE, and the Cochrane Library databases. Abstracts of articles reporting the outcomes of the harmonic scalpel and traditional surgical procedures in neck dissection were selected. The MEDLINE database was searched with the following search terms as keywords: (a) “harmonic scalpel” and (b) “neck dissection” (medical subject heading, or MeSH). The EMBASE and Cochrane databases were searched by using (a) “neck dissection” and (b) “harmonic scalpel” as text words. Reference lists within the retrieved articles were used as secondary reference sources.

### Inclusion criteria

Studies were included if they fulfilled all the following inclusion criteria:(a) patients who were diagnosed with head and neck cancer undergoing neck dissection without any treatment before surgery; (b) the article must compare the outcomes of the harmonic scalpel and traditional hemostasis in neck dissection; (c) outcome measures must include the operative time or intraoperative bleeding; (d) randomized controlled trials without limitations on language and publication status; and (e) summary data available for the outcomes of interest.

### Exclusion criteria

Studies were excluded if they were non-randomized. Case reports, letters to the author, comments and reviews were excluded.

### Quality assessment and data analysis

The quality and risk of bias of all the included trials were independently assessed by the two reviewers, ZHR and JLX, based on the recommendations from the Cochrane Handbook of Systematic Review of Interventions. The criteria included random sequence generation, allocation concealment, blinding for participants and personnel, blinding of outcome assessments, incomplete outcome data, selective outcome reporting, and other biases. An assessment of the risk of bias was categorized as ‘Low risk of bias’, ‘Unclear risk of bias’, or ‘High risk of bias’ in each domain, based on the guidelines from the Cochrane Handbook, with notes explaining the specific reasons for each categorization in the risk of bias table Any conflicts in opinion were resolved by discussion.

We extracted data for the studies using a standardized data extraction form (Review Manager 5.3). We attempted to contact the study authors for any relevant missing or unclear data. We also asked the authors to confirm whether the study was duplicated and whether there was any doubt if the studies shared the same patients. We excluded the studies that did not meet the inclusion criteria in terms of study design. One reviewer (ZHR) extracted the data, which was checked by another reviewer (JLX). Differences in opinion were resolved by discussion.

### Statistical analysis

All the individual outcomes were pooled using RevMan5.3 (Cochrane Collaborative, Oxford, England). The mean differences (MD) were calculated for the operative time, intraoperative bleeding, the amount of drainage, and hospital stay. The outcomes were aggregated and analyzed using a random-effect model. Statistical heterogeneity, defined as the variation in results between the studies, was assessed by using the Chisquared distributed Q statistic. When the statistical heterogeneity was great, subgroup analyses was needed. Subgroup analyses and sensitivity analyses were performed to assess whether there was a difference in the operative time and intraoperative bleeding.

This meta-analysis followed the Preferred Reporting Items for Systematic Reviews and Meta-Analysis (PRISMA) statement guidelines, and the relevant checklist can be found as S1 PRISMA Checklist.

## Results

### Search findings

**Fig 1:**
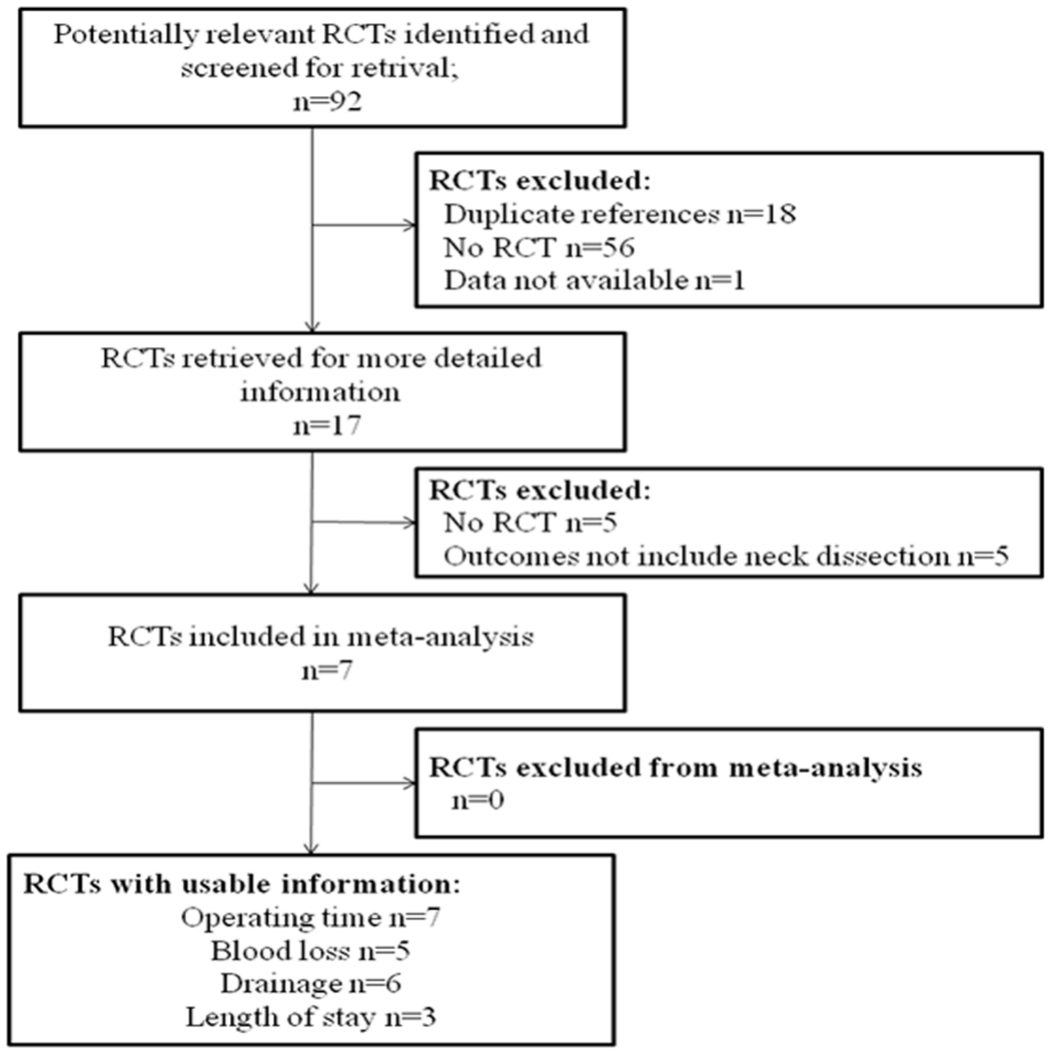
Flow diagram of literature search.

Our literature review identified 92 articles that involved potentially relevant RCTs; 75 were excluded because they were not actually RCTs (n = 56), had duplicate references (n = 18) or because of data not available (n = 1). The remaining 17 articles were further assessed for eligibility and another 10 articles were eventually excluded. Fig 1 shows the flowchart of studies retrieved and excluded and lists the reasons for their exclusion. Seven studies comprising a total 406 neck dissection cases were included in this meta-analysis, including 201 in the harmonic scalpel group. (The information of these 7 studies is in Table 1.)

**Table 1:** The information of these 7 included studies.

Author and year of publication	Location of study	Sample size	Primary outcomes	Inclusion criteria (exclusion criteria)
Dean et al. 2014 [11]	Spain	63	Surgical time; surgical blood loss; drain time; drainage fluid volume of first 3 days.	Patients with squamous cell carcinoma (SCC) of the oral cavity without any treatment before surgery.
Ferri et al. 2013 [8]	Italy	61	Operative time; intraoperative blood loss; drainage fluid volume of first 2 days; postoperative pain; hospital stay; shoulder syndrome.	Inclusion criteria: age >18 years old; acceptance to participate in the study; scheduled neck dissection (ND) with Primary head and neck SCC. Exclusion criteria: preoperative medication or irradiation; coagulation disorders; pregnancy; case in which the ND specimen could not be separated from the primary tumor.
Koh et al. 2008 [12]	Republic of Korea	65	Operating time; grade of intraoperative bleeding; drain time; duration of hospital stay.	Patients with thyroid papillary carcinoma; and excluding patients who required lateral compartment ND or mediastinal dissection for preexisting lymph node metastasis or had clinical or laboratory indicators of coagulation disorders.
Miccoli et al. 2009 [15]	Italy	37	Operative time; drainage volume of 24hours and 48hours.	Patients with the diagnosis of papillary thyroid carcinoma and lymph node metastases in the lateral compartment.
Salami et al. 2008 [16]	Italy	20	Duration of operation; intraoperative blood loss of first 3 days	Patients with laryngeal carcinomas, extended up to the base of the tongue; cervical metastatic lymph nodes at levels II, III, and IV; and a negative evaluation for metastatic disease.
Shin et al. 2013 [7]	Republic of Korea	59	Operative time; intraoperative blood loss; suction drainage amount; drainage duration.	Inclusion criteria: age >18 years old; the preoperative diagnosis of all the patients was HNSCC; only tumors originating from the oral cavity, oropharynx, hypopharynx, and larynx. Exclusion criteria: the ND specimen could not be separated from the primary tumor.
Walen et al. 2011 [14]	Canada	34	Operative time; intraoperative blood loss; vascular complications; neurologic complications; drainage fluid volume of first 48 hours and 1 week; hospital stay.	Inclusion criteria: age >18 years old; HNSCC need a levels I–IV ND. Exclusion criteria: if there was any prior treatment for head and neck cancer or if they were unwilling or unable to give informed consent.

### Methodological quality of the included studies

**Fig 2:**
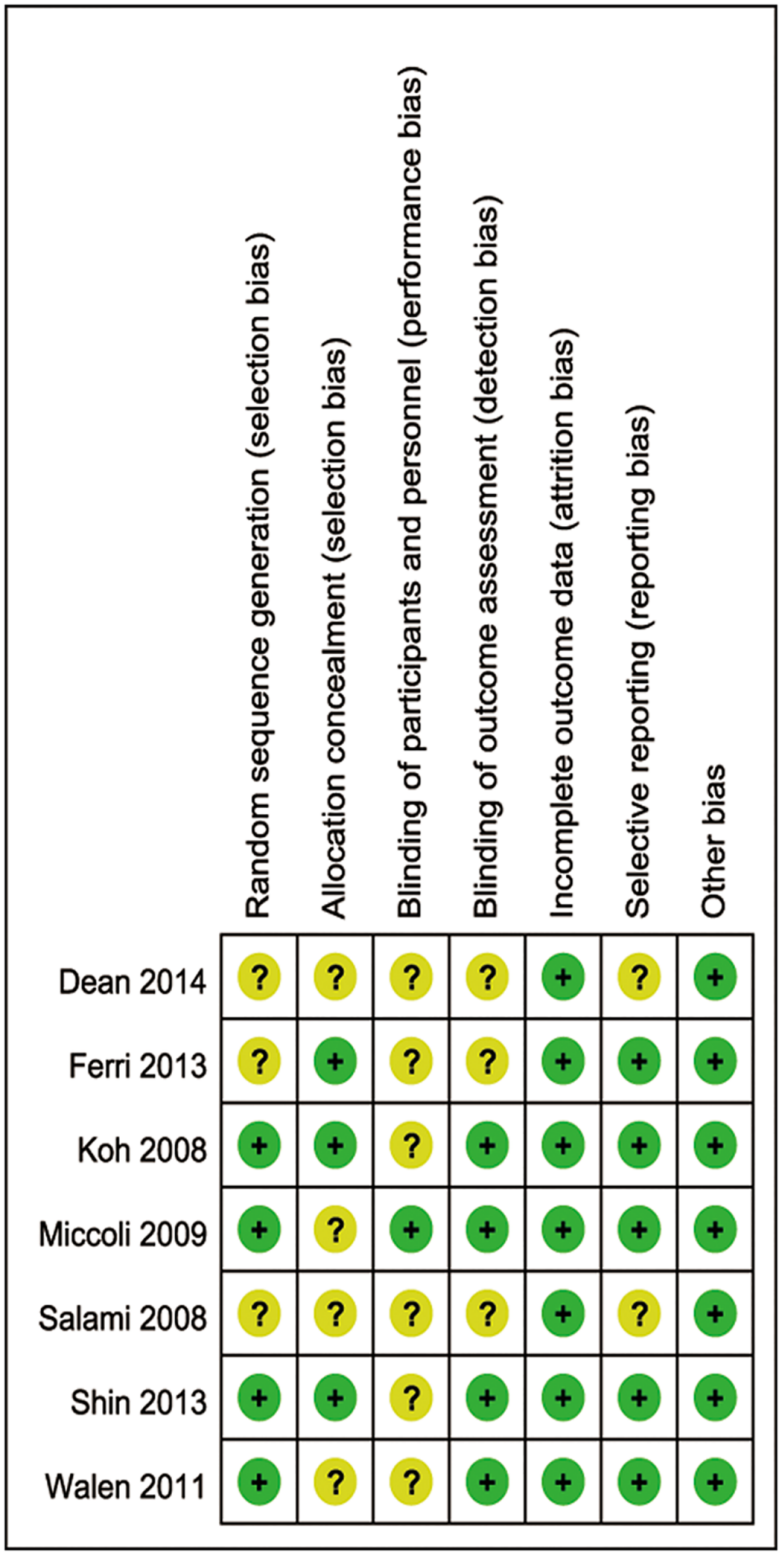
Risk of bias summary. Two studies were identified as being of a higher design quality. Three studies were identified as being of a lower design quality. The quality of the remaining two studies was moderate. (“+”means the bias is low risk, “?” means the bias is unclear).

The studies by Koh [12], Miccoli [15] and Shin et al. [7] were identified as being of a higher design quality. The studies by Dean [11] and Salami et al. [16] were identified as being of a lower design quality because neither comprised blinded participants or an outcome assessment, and the use and reporting of random sequence generation and allocation concealment may have been inadequate. In addition, the quality of the studies by Ferri [8] and Walen et al. [14] was moderate (Fig 2). Overall, the agreement between the two assessors about the quality of the seven studies was moderate or high, although there was still some controversy. When there was controversy in the assessment of the quality of the studies, a third person was asked to review the study. According to the Funnel plot, there is an unconspicuous asymmetry (S1 Fig). That is to say, our study has a little reporting bias.

### Meta-analysis

#### Operative time

**Fig 3:**
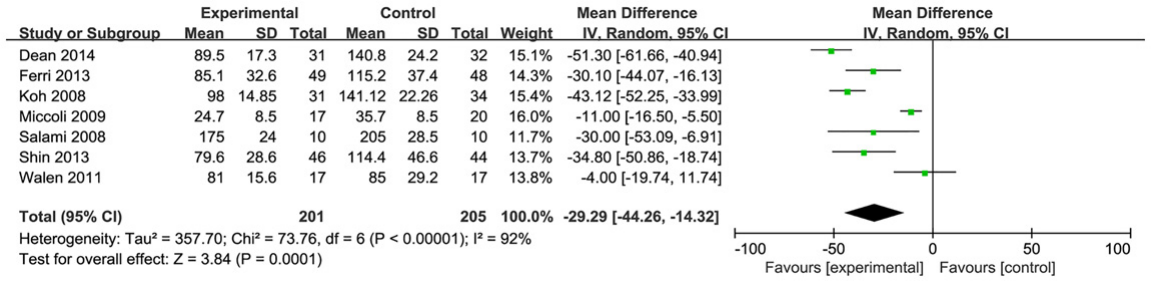
Forest plot for operative time. The operative time with the harmonic scalpel was significantly shorter than that with conventional hemostasis, but the statistical heterogeneity was unacceptably large (I^2^ = 92%).

**Fig 4:**
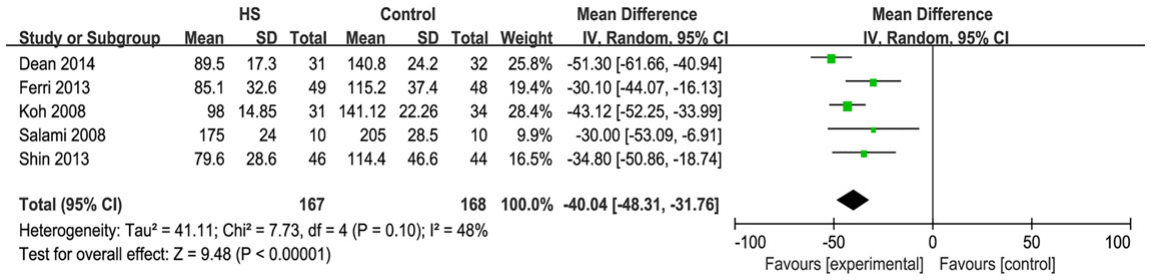
Forest plot for the operative time (sensitivity analysis). Heterogeneity became acceptable (I^2^ = 48%) and the effect measure remained significant (P<0.00001).

Seven studies reported the operative time. When these studies were quantitatively combined, the mean difference in the operative time for neck dissection was 29.3minutes shorter using the harmonic scalpel compared with conventional hemostasis [mean difference: -29.29; 95% CI = (-44.26, -14.32); P = 0.0001] (Fig 3). However, there was great statistical heterogeneity that was driven by two studies (Walen [14] and Miccoli et al. [15]). When these two studies were excluded, the heterogeneity became acceptable (I^2^<50%) and the effect measure remained significant [I^2^ = 48%; mean difference: -40.04; 95% CI = (-48.31, -31.76); P<0.00001] (Fig 4).

The operative times may have been affected by whether the skin flaps were completely raised or not; as Walen et al. [14] discussed in their paper, the duration of surgery were shorter in both the harmonic scalpel and conventional hemostasis groups. The skin incisions and surgical method used in Miccoli’s study [15] was different from those used in the study by Walen et al.

#### Intraoperative bleeding

**Fig 5:**
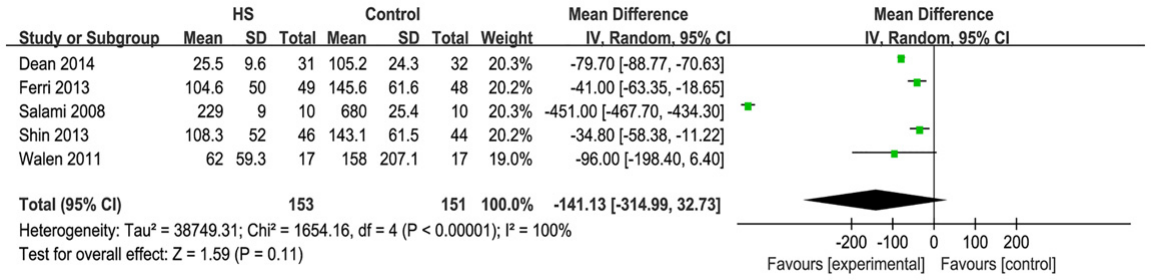
Forest plot for blood loss. The intraoperative blood loss with the harmonic scalpel was shorter than that with conventional hemostasis, but not significantly, and the statistical heterogeneity was unacceptably large (I^2^ = 100%).

**Fig 6:**
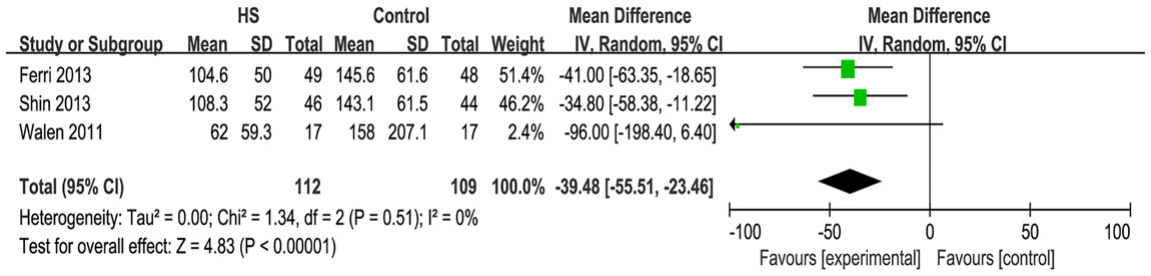
Forest plot for blood loss(sensitivity analysis). Heterogeneity became acceptable (I^2^ = 0%) and the effect measure was significant(P<0.00001).

Five studies reported on intraoperative bleeding. When these studies were quantitatively combined, the mean difference in the intraoperative blood loss during the neck dissection was 141.1 milliliters less using the harmonic scalpel compared with conventional hemostasis [mean difference: -141.13; 95% CI = (-314.99, 32.73); P = 0.11] (Fig 5). However, the effect measure was not significant, and there was great statistical heterogeneity that was driven by two studies (Dean [11] and Salami et al. [16]). When these two studies were excluded, heterogeneity became acceptable and the effect measure was significant [I^2^ = 0%; mean difference: -39.48; 95% CI = (-55.51, -23.46); P<0.00001] (Fig 6).

The studies of Dean et al. and Salami et al did not describe the methods used to record the intraoperative bleeding. These two studies were initiatedbefore2005 and other studies were started after 2005.

#### Amount of drainage

**Fig 7:**
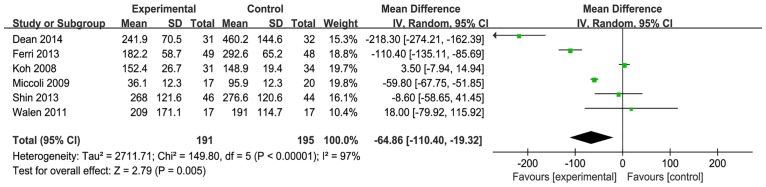
Forest plot for the amount of drainage. Total drainage fluid volume with the harmonic scalpel was significantly shorter than that with conventional hemostasis but the statistical heterogeneity was unacceptably large (I^2^ = 97%).

**Fig 8:**
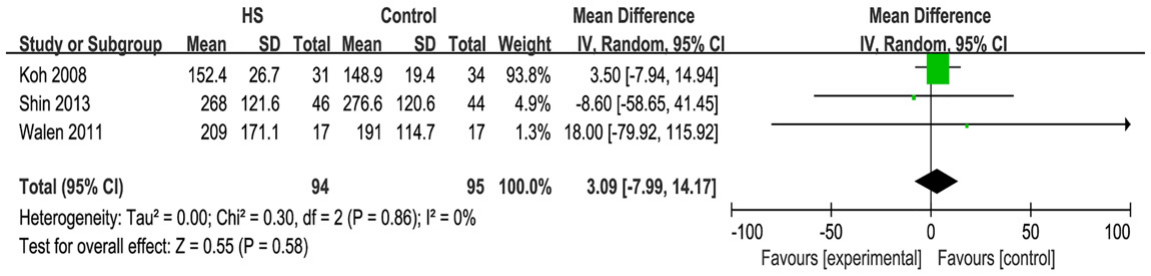
Forest plot for the amount of drainage (sensitivity analysis). Heterogeneity became acceptable (I^2^ = 0%) but the effect measure was not significant (P = 0.58).

Six studies reported on the amount of drainage. When the six studies were quantitatively combined, the mean difference in the total drainage fluid volume for the neck dissection was 64.9 milliliters less using the harmonic scalpel compared with conventional hemostasis [mean difference: -64.86; 95% CI = (-110.40, -19.32); P = 0.005] (Fig 7). However, the statistical heterogeneity was great and was driven by three studies (Ferri [8], Dean [11] and Miccoli et al. [15]). When these three studies were excluded, the heterogeneity and the effect measure were not significant [I^2^ = 0%; mean difference: 3.09; 95% CI = (-7.99, 14.17); P = 0.58] (Fig 8).

Various methods were used to record the amount of drainage in these studies. Moreover, the recording time ranged from 2 to 7 days.

#### Hospital stay

**Fig 9:**
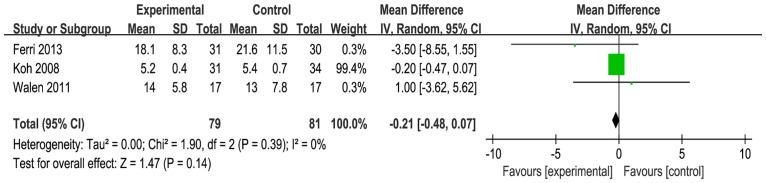
Forest plot for the hospital stay. Heterogeneity was acceptable (I^2^ = 0%). However, the difference between the two groups was not significant (P = 0.14).

Three studies reported on the hospital stay. When the three studies were quantitatively combined, the mean difference in the hospital stay following a neck dissection was 0.2 days less using the harmonic scalpel compared with conventional hemostasis [mean difference: -0.21; 95% CI = (-0.48, 0.07); P = 0.14] (Fig 9). The heterogeneity was acceptable but the effect measure was not significant.

Finally, these studies conducted descriptive research on perioperative complications, and we did not observe any serious complications using the HS during ablation. Moreover, the overall perioperative complications after neck dissection did not differ significantly between the 2 groups.

## Discussion

The HS was introduced in the early 1990s. Many recent articles have described the advantages of the HS in neck surgery [17–20]. In particular, there have been many studies demonstrating that the HS reduces the operative time, has benefits in hemostasis and is minimally invasive in neck dissection [14–16, 21]. However, there is still some controversy about these procedures [11, 14]. In this meta-analysis, we prospectively assessed whether the HS has benefits in terms of hemostasis and being minimally invasive, checking the foremost outcomes (i.e., operative time, intraoperative bleeding, total drainage fluid volume, and length of hospital stay) by comparing the harmonic scalpel method versus conventional hemostasis. To reduce any potential bias, we developed a detailed protocol before initiating the study, performed a meticulous search for published studies and used explicit methods for data selection, data analysis, and data extraction.

This meta-analysis confirmed the impact of the HS on the operative time. There was a reduction in the mean operative time for the HS of 29.29 minutes (which is greater than 20%) when compared with conventional surgical methods; this result is statistically significant. However, there was great statistical heterogeneity, driven by the studies of Walen et al. and Miccoli et al. When these two studies were excluded, the heterogeneity became acceptable and the effect measure remained significant. There was a mean operative time reduction for the HS of 40.04 minutes when compared with conventional hemostasis. In the study by Walen et al., the subplatysmal skin flaps were completely raised before the operative time was recorded. In the other study by Miccoli et al., the operative time was recorded from the time of the first cutaneous incision. Neck dissection in Miccoli’s study was conducted centrally; therefore, the skin incisions and surgical method in Miccoli’s study were different from those in Walen’s study. The operative time in Miccoli’s study was much shorter than that in Walen’s study. This could explain why there was statistical heterogeneity between these two and other studies.

This meta-analysis also confirmed the impact of the HS on intraoperative bleeding. There was a mean blood loss reduction for the HS of 141.13 milliliters. However, the effect measure was not significant and statistical heterogeneity was unacceptably large. When these two studies that were the main cause of the heterogeneity were excluded, the heterogeneity became acceptable and the effect measure became significant. There was a mean blood loss reduction for the HS of 39.48 milliliters. These two studies did not describe the methods used to record the intraoperative bleeding. The two studies began before 2005 and other studies were initiated later than 2005. This could explain the statistical heterogeneity between these two studies and others.

There were various methods of recording the amount of drainage in these studies and the recording time ranged from 2 to 7 days. The statistical heterogeneity was unacceptably large and therefore the finding of the amount of drainage in this meta-analysis had a low credibility. More studies are required to substantiate our findings.

Safety is a major concern in neck dissection. Our systematic review did not reveal any differences for the HS compared with conventional surgical techniques. This finding is consistent with the more general systematic review of Matthews et al [22], which refers to the use of the HS for various procedures.

The results for the length of hospital stay were only based on three studies. The mean difference in the hospital stay was 0.2 days less using the harmonic scalpel compared with conventional hemostasis but the difference was not significant. Further studies are required to substantiate our findings.

Every systematic review, including our own, has its limitations. First and foremost, the number of included studies was low at only seven, and the number of cases was also small. More studies are required to further confirm our findings. Second, several studies provided only limited information on blinding, randomization, and allocation concealment to allow a judgment of whether the randomized controlled trials (RCTs) were conducted properly. Finally, there was unacceptably great heterogeneity among these studies. These all have an impact on the outcomes measured and might have influenced the results.

## Conclusion

The systematic review showed that there is clear evidence that using the harmonic scalpel for neck dissection significantly reduces the operative time and drainage fluid volume and that it is not associated with an increase in the length of hospital stay or perioperative complications. Therefore, the harmonic scalpel is a safe and effective method for neck dissection. However, the small sample size available for this systematic review limited the power of this quantitative meta-analysis. Furthermore, the statistical heterogeneity was high. In addition, we can’t determine whether the harmonic scalpel can reduce intraoperative bleed loss during neck dissection now. It may therefore be too early to place complete confidence in these results. Further studies are required to substantiate our findings.

## Supporting Information

S1 Fig Forest plot of this meta-analysis. According to the Funnel plot, there is an unconspicuous asymmetry.
(TIF)

S1 PRISMA Checklist PRISMA Checklist for this meta-anlysis. (DOC)
