# Supplementary figures and images for: The Harmonic Scalpel versus Conventional Hemostasis for Neck Dissection: A Meta-Analysis of the Randomized Controlled Trials
Source: PLoS One. 2015 Jul 10;10(7):e0132476. doi: 10.1371/journal.pone.0132476 (PMC4498925; doi:10.1371/journal.pone.0132476)

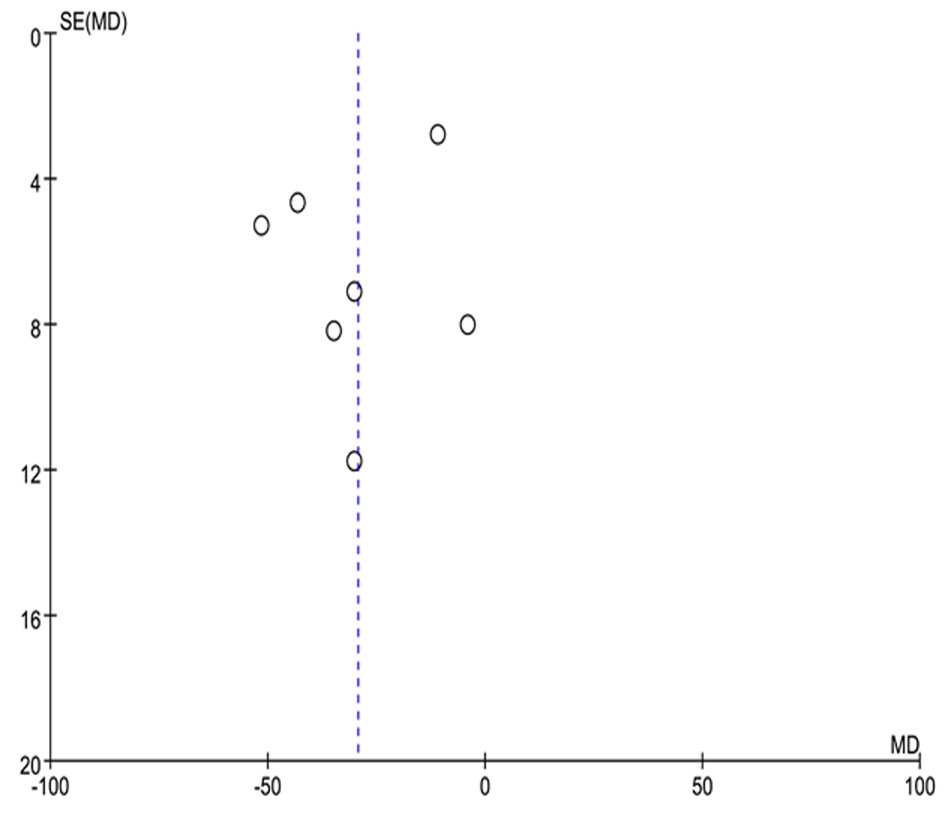

Supplement: S1 Fig — According to the Funnel plot, there is an unconspicuous asymmetry. (TIF) [file pone.0132476.s001.tif]
